# Metabolic syndrome severity score in the middle-aged and elderly Iranian population: A cross-sectional survey of Bandare-Kong Cohort Study (the findings of PERSIAN Cohort Study)

**DOI:** 10.3389/fpubh.2022.1010735

**Published:** 2023-01-06

**Authors:** Amin Ghanbarnejad, Masoumeh Kheirandish, Feysal Yousefzade, Arash Rahimi, Abnoos Azarbad, Azim Nejatizadeh, Mehdi Shahmoradi

**Affiliations:** ^1^Endocrinology and Metabolism Research Center, Hormozgan University of Medical Sciences, Bandar Abbas, Iran; ^2^Social Determinants in Health Promotion Research Center, Hormozgan University of Medical Sciences, Bandar Abbas, Iran; ^3^Student Research Committee, Faculty of Medicine, Hormozgan University of Medical Sciences, Bandar Abbas, Iran; ^4^Molecular Medicine Research Center, Hormozgan Health Institute, Hormozgan University of Medical Sciences, Bandar Abbas, Iran

**Keywords:** metabolic syndrome, metabolic syndrome severity score, Prospective Epidemiological Research Studies in IrAN (PERSIAN), middle-age, adult

## Abstract

**Background:**

Metabolic syndrome (MetS) is defined as the presence of several metabolic risk factors. The traditional MetS criteria have been considered insufficient for evaluating individuals at risk. MetS has always been categorized using binary criteria, which deny that the risk associated with MetS is likely to exist as a continuum. Also, MetS may present differently depending on age, sex, race, or ethnicity. We aimed to derive age-sex-specific equations for MetS severity scores within a southern Iranian population.

**Methods:**

This study used first-phase data from the Bandare-Kong Non-Communicable Diseases (BKNCD) Cohort Study as part of the Prospective Epidemiological Research Studies in IrAN (PERSIAN). After exclusion of the pregnant women, diabetic patients, and individuals taking antihypertensive, antihyperlipidemic, and antidiabetic medications, 2,735 individuals aged 35 to 70 years were selected for analysis. The diagnosis of MetS was based on the National Cholesterol Education Program (NCEP) criteria for the Iranian population. Confirmatory factor analysis (CFA) was performed to formulate MetS severity scores. The receiver operating characteristic (ROC) analysis was performed to validate MetS severity score equations for age-sex-specific categories.

**Results:**

Triglyceride had the highest factor loading range in all age-sex categories for determining the MetS severity score. Conversely, systolic blood pressure and fasting plasma glucose (FPG) exhibited the lowest factor loadings across all age-sex groups. In both sexes, when age was considered, systolic blood pressure and FPG factor loadings were less significant among subjects aged ≥45 and 35–44 years, respectively.

**Conclusion:**

MetS severity scores might be more applicable than the current criteria of MetS. Prospective population-based studies should be conducted to assess the accuracy and validity of the MetS severity score for predicting cardiometabolic diseases.

## Introduction

Several cardiometabolic risk factors are embedded in the definition of metabolic syndrome (MetS). Abdominal obesity, hypertriglyceridemia, high blood pressure, and low serum high-density lipoprotein (HDL) levels are the major components of MetS (1). Epidemiological studies suggest that the incidence of type 2 diabetes mellitus (T2DM) and cardiovascular disease (CVD) is increased by 5- and 2-folds, respectively in individuals with MetS (2, 3). The results of a systematic review and meta-analysis showed a 30.4% prevalence of MetS in the Iranian population (4). According to the national study of MetS prevalence in Iran, prevalence rates ranged from 32 to 47.6%, depending on the criteria used (5). Furthermore, the Bandare-Kong Non-Communicable Diseases (BKNCD) Cohort Study revealed a 34.5% MetS prevalence (6).

Evidence suggests that the traditional MetS criteria cannot accurately predict the risk of cardiometabolic disorders due to racial and ethnic differences and are not applicable to high-risk individuals (7–15). Moreover, MetS has always been categorized using binary criteria, which deny that the risk associated with MetS is likely to exist as a continuum (16, 17). Furthermore, it may be difficult to monitor changes in MetS status over time since its dichotomous status might vary based on whether the component cut-offs are satisfied at each follow-up (16, 18, 19). This study aimed to determine MetS severity score formulas in a southern Iranian population in light of the increasing prevalence of chronic diseases such as T2DM and CVD and the limitations of traditional criteria.

## Methods

### Participants

The BKNCD provided the data for this cross-sectional study (20). The study included 4,063 individuals aged 35 to 70 recruited from Hormozgan province in southern Iran from 2016 to 2018 as part of the Prospective Epidemiological Research Studies in IrAN (PERSIAN). Pregnant women, individuals with diabetes, and individuals taking antihyperlipidemic, antihypertensive, or antidiabetic medications were not included, leaving a total of 2,735 participants for the final analysis.

### Study design

The Ethics Committee of the Hormozgan University of Medical Sciences approved this study (ethics code: IR.HUMS.REC.1399.514), which is in compliance with the statements of the Declaration of Helsinki. Informed consent was provided by all subjects. Sociodemographic data were collected using face-to-face interviews by trained interviewers.

A standard mechanical scale was used for weight measurement (to the nearest 0.5 kg), while subjects wore no shoes and very little clothing. Height measurements were performed while subjects were barefoot and with relaxed shoulders. The average of two waist circumference (WC) measurements were recorded for each subject. WC was measured as the circumference approximately midway the inferior border of the last detectable rib and top of the iliac crest in the mid-axillary line parallel to the floor. A mercury sphygmomanometer with a suitable cuff-size was used for blood pressure (BP) measurements (21), which was done after at least a 5-min rest and while the subject was sitting with his/her arms at the level of the heart and feet on the floor. The measurements were repeated for each subject with at least a 5-min interval and the average was recorded.

Venous blood samples taken after an 8-h overnight fasting were used for fasting plasma glucose (FPG) measurements by the glucose oxidase technique. Venous blood samples taken after a 12-h overnight fasting were used to measure triglyceride (TG) and HDL levels with an enzymatic method. The reason for a longer fasting period for the measurement of these lipid components was to prevent their overestimation (22). According to the Iranian obesity association guidelines, WC ≥95 cm was defined as central obesity. The Iranian National Committee on Obesity has recommended the following MetS criteria and cut-off values, based on the National Cholesterol Education Program (NCEP) criteria, with a person qualifying for MetS if they met at least three of the five components (23):

1. WC ≥ 95 cm.
2. FPG ≥ 100 mg/dl or drug treatment for elevated blood glucose.
3. HDL < 40 mg/dl in men, <50 mg/dl in women, or drug treatment for low HDL.
4. TG ≥ 150 mg/dl or taking lipid-lowering agents.
5. BP ≥ 130/85 mmHg or taking antihypertensive medications.

### Data analysis

The SPSS (IBM Corp. 2017. IBM SPSS Statistics for Windows, Version 25. Armonk, NY: IBM Corp) software was used for statistical analyses, the EQS (Bentler, P. M. 2006. EQS 6 Structural Equations Program. Encino, CA: Multivariate Software, Inc.) was used for confirmatory factor analysis (CFA).

A CFA approach was used to calculate MetS severity scores for all standard MetS components to determine each component's weighted contribution to a latent age- and sex-specific MetS factor. Systolic blood pressure (SBP) was used to represent BP. TG had a skewed distribution; therefore, the log-transformed variable of TG was used in the analysis. We used inversed HDL to achieve higher factor loading in order to be interpreted similar to other variables. Standardization was performed on the entire sample [mean = 0; standard deviation (SD) = 1].

CFA was designed as a one-factor model, with the assumption that the errors of measurement were not correlated among the five components. Factor loadings illustrated the strength of association between the underlying MetS factor and each component. Factor loadings >0.3 were considered clinically significant.

The maximum likelihood method was used to estimate the model parameters. Since the χ^2^ test is sensitive to large sample sizes and is significant in most cases; other fit indices were examined, such as the goodness of fit index (GFI, ≥0.90 indicates a good fit), the Beetle-Browed normed fit index (NFI, ≥0.90 indicates a good fit), and the McDonald fit index (MFI, ≥0.90 indicates a good fit). Additionally, the comparative fit index (CFI, ≥0.90 indicates a good fit), root means squared error of approximation (RMSEA, <0.05 indicates a good fit, values around 0.08 represent good fit), and the standardized root mean square residual (SRMR, ≤0.08 indicates good fit) were measured for model assessment.

Based on standardized factor coefficients from the model, MetS severity scores were calculated for each individual. By taking appropriate linear combinations of the variables in the analysis, factor scores (estimates of an individual's latent factor) can be calculated. The factor score and MetS severity scores were estimated by back-transforming the five components to the unstandardized forms. The estimated MetS severity score can be interpreted as a Z-score, with higher scores indicating increased MetS risk. In order to test the MetS severity Z-score's ability to discriminate against Iranian NCEP criteria for MetS, a receiver operating characteristic (ROC) curve was drawn.

Due to the large sample size, all reported Chi-square tests were significant. Plus, it was not considered as a criterion of model fitting. GFI, adjusted GFI, MFI, and SRMR ranged between 0.96–0.98, 0.90–0.98, 0.95–0.98, and 0.042–0.071, respectively, indicating good model fitting. However, CFI, NFI, and RMSEA failed to reach acceptable cutoff values except for females aged 35–44 years. Therefore, based on the indices mentioned, each sex/age group's resulting pattern was considered a reasonable model for the studied sample.

## Results

After applying the exclusion criteria, 2,735 individuals were finally selected for CFA. The CFA of MetS components in this study resulted in producing a model that perfectly fits the data (Table 1).

**Table 1:** Confirmatory factor analysis.

	**Total**	**Male**	**Female**	**Male, 35–44 yr**	**Male, ≥45 yr**	**Female, 35–44 yr**	**Female, ≥45 yr**
**No**.	2,735	1,262	1,473	635	627	782	691
**Fit indices**							
**GFI**	0.98	0.97	0.98	0.96	0.97	0.98	0.98
**SRMR**	0.055	0.064	0.05	0.071	0.063	0.042	0.051
**AGFI**	0.94	0.90	0.94	0.88	0.90	0.95	0.94
**MFI**	0.98	0.96	0.98	0.95	0.96	0.98	0.98
**CFI**	0.86	0.81	0.87	0.76	0.82	0.92	0.81
**NFI**	0.86	0.81	0.86	0.76	0.81	0.90	0.79
**RMSEA (90% CI)**	0.096 (0.082–0.110)	0.118 (0.097–0.139)	0.090 (0.070–0.110)	0.139 (0.110–0.169)	0.113 (0.084–0.144)	0.075 (0.048–0.103)	0.085 (0.058–0.116)
**Chi-square (df)**	133.75 (5)	98.27 (5)	70.01 (5)	64.81 (5)	52.02 (5)	29.05 (5)	31.64 (5)
**Factor loadings**							
**WC**	0.32	0.50	0.49	0.54	0.53	0.55	0.32
**SBP**	0.24	0.19	0.35	0.33	0.16	0.34	0.22
**HDL**	0.42	0.48	0.31	0.48	0.45	0.38	0.36
**FPG**	0.25	0.23	0.34	0.27	0.23	0.30	0.25
**TG**	0.84	0.72	0.68	0.64	0.74	0.71	0.67

GFI, goodness of fit index; SRMR, standardized root mean square residual; AGFI, adjusted goodness of fit index; MFI, McDonald fit index; CFI, comparative fit index; NFI, normed fit index; RMSEA, root means squared error of approximation; WC, waist circumference; SBP, systolic blood pressure; HDL, high-density lipoprotein; FPG, fasting plasma glucose; TG, triglyceride.

The factor loading of TG ranged from 0.64 to 0.74 among all sex-age categories, as shown in Table 1. In contrast, FPG and SBP had the lowest factor loadings. In both sexes aged ≥45 years, the factor loading of SBP was <0.3; this component had less importance in determining the MetS severity score in these groups. Also, FPG had factor loadings <0.3 across all age-sex groups.

### Equations for calculating MetS severity Z-score

Table 2 provides the equations for calculating the severity Z-score for MetS based on clinical components. These formulas are based on actual values rather than standardized measures, produced by back-transformation of coefficients from standardized factor analysis. Table 2 compares the MetS severity Z-score formulas based on different age- and sex-specific categories. Based on these equations, MetS severity score in the population could be calculated.

**Table 2:** Equations for sex- and age-specific metabolic syndrome risk Z-score.

**Sex**	**Age**	
Male	35–44	−9.6162 + 1.3278^*^ln (TG) + 0.0112^*^SBP + 0.0204^*^WC-0.0312^*^HDL + 0.0176^*^FPG
	≥45	−9.7368 + 1.3175^*^ln (TG) + 0.0116^*^SBP + 0.0208^*^WC-0.0296^*^HDL + 0.0178^*^FPG
Female	35–44	−9.8570 + 1.3271^*^ln (TG) + 0.0112^*^SBP + 0.0199^*^WC-0.0267^*^HDL + 0.0186^*^FPG
	≥45	−9.7675+1.2913^*^ln (TG)+0.0114^*^SBP+0.0200^*^WC-0.0243^*^HDL+0.0178^*^FPG

TG, triglyceride; SBP, systolic blood pressure; WC, waist circumference; HDL, high-density lipoprotein; FPG, fasting plasma glucose.

**Figure 1:**
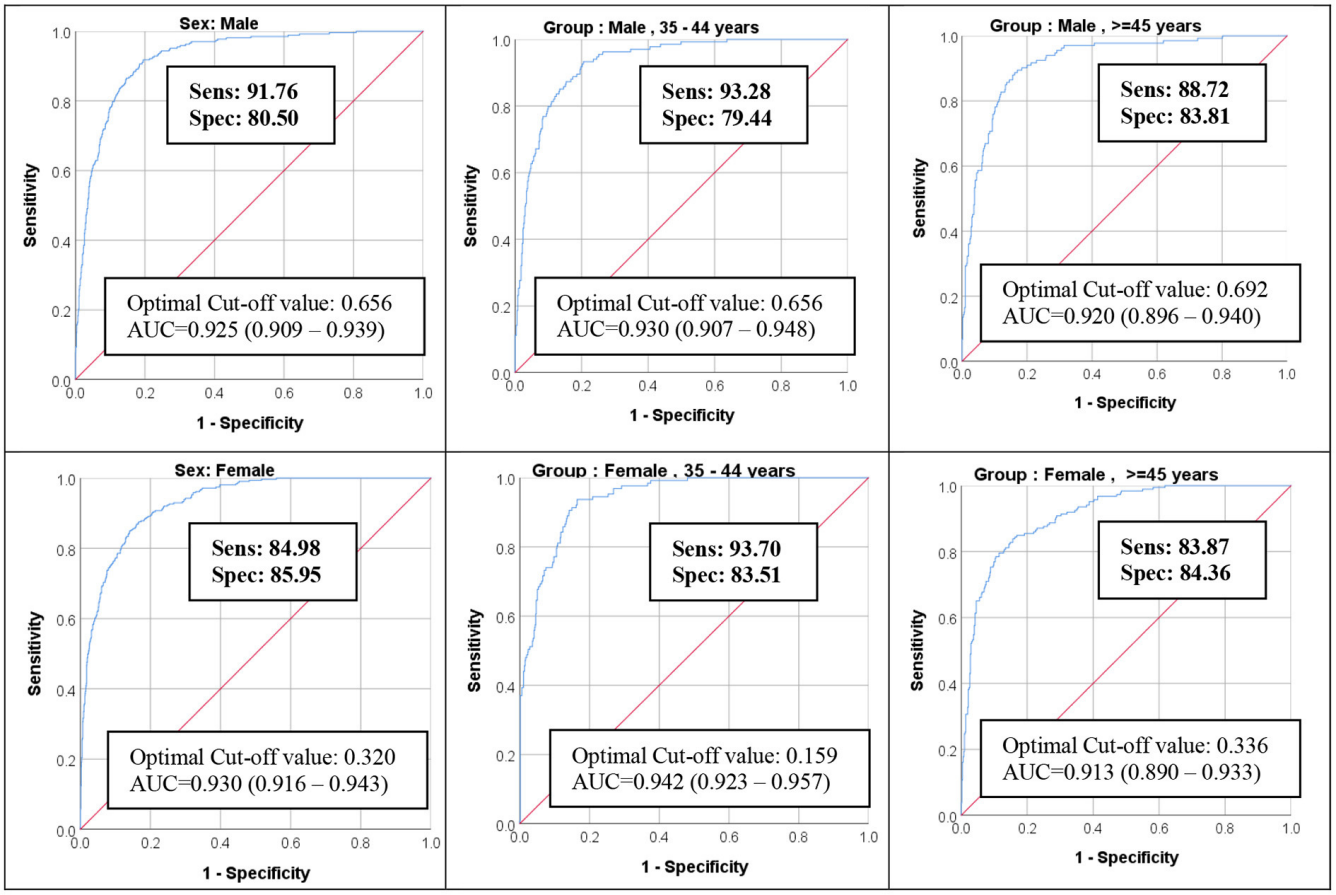
ROC Curve of MetS severity Z-score for predicting MetS NCEP Criteria for the Iranian population. ROC, receiver operating characteristic; MetS, metabolic syndrome; AUC, area under the curve.

ROC analysis of the resulting risk score was excellent to predict Iranian NCEP classification (Figure 1). The area under the curve (AUC) values of MetS severity score ROC curves were ≥0.91 for every age-sex group.

## Discussion

The present study used CFA to calculate MetS severity scores for the middle-aged and elderly population who participated in the BKNCD cohort study. The results indicated that TG had the highest, while SBP and FPG had the lowest factor loadings for calculating MetS severity scores. Among males and females aged ≥45 years, factor loading of the SBP component had less importance in determining the MetS severity score. Compared to other age and sex groups, FPG had a minor factor loading effect among males and females aged 35–44 years.

Studies have shown that traditional MetS criteria alone cannot accurately predict the risk of progression to major non-communicable diseases such as CVD and T2DM. For instance, non-Hispanic and Hispanic whites exhibit a higher prevalence of MetS compared to non-Hispanic blacks; however, the black population has higher insulin resistance rates, T2DM, and CVD mortality rates. Consequently, there is a concern regarding the potential applicability of traditional MetS criteria to determine the cardiometabolic risk and predict the risk of CVD and T2DM. Essentially, the traditional criteria could only identify individuals with three or more abnormal elements of MetS that are not even necessarily risk factors for CVD and T2DM. Therefore, it is likely that individuals with MetS measurements below the threshold in all five components will be at greater risk than those with measurements just above the threshold in three MetS components but low or normal measurements in the other two (7–16, 24).

Furthermore, it is impossible to follow individuals over time in terms of changes in MetS status using the traditional criteria (18, 19). A study conducted in the US assessed the traditional criteria for MetS with regard to sex and different racial-ethnic groups in order to calculate MetS severity. According to this study, insulin resistance and other biomarkers, including high sensitivity C-reactive protein and uric acid, were associated with MetS severity scores. It is important to note that even though the prevalence of MetS by traditional criteria in non-Hispanic black men with diabetes was reported low, this group scored highly on the severity of MetS. This highlights the limitations of the old method for identifying high-risk individuals and demonstrates the effectiveness of the newly developed method for assessing MetS. According to that study, a clinically-accessible and interpretable measure of MetS was provided by the equations designed to score MetS severity, which may prove useful in detecting adults at higher risk of MetS-associated diseases and measuring changes within individuals over time (25, 26). Furthermore, the effectiveness of this method has been evaluated in Australia, South Korea, and Singapore (27–29).

A major strength of this study was that it was based on data obtained from a well-designed, population-based cohort study. A primary focus was also on verifying the scoring system from this study using the modified NCEP criteria for Iranians in the definition of MetS. This study had some limitations: the sample size was not very large, and the study population was restricted to southern Iranians. Additionally, confirmatory factor analysis was conducted on a relatively healthy group of individuals without medication usage to ensure that MetS-related measures would not be affected. Also, this study did not include individuals under 35 and over 70 years of age. Therefore, the results could not be generalized to all age groups.

## Conclusion

MetS severity scores might more accurately identify at-risk individuals than the existing MetS criteria. There is a need to conduct a prospective study with a large population of participants to evaluate the accuracy and validity of the MetS severity score for predicting cardiometabolic diseases.

## Data availability statement

The raw data supporting the conclusions of this article will be made available by the authors, without undue reservation.

## Ethics statement

The studies involving human participants were reviewed and approved by Ethics Committee of Hormozgan University of Medical Sciences, Bandar Abbas, Iran. The patients/participants provided their written informed consent to participate in this study.

## Author contributions

AG performed a comprehensive statistical analysis. MK designed and wrote the manuscript. FY wrote the manuscript. AN supervised the study. AR and AA revised the manuscript. MS interpreted the data. All authors read and approved the final manuscript.
